# Mutual information spectrum for selection of event-related spatial components. Application to eloquent motor cortex mapping

**DOI:** 10.3389/fninf.2013.00053

**Published:** 2014-01-20

**Authors:** Alexei Ossadtchi, Platon Pronko, Sylvain Baillet, Mark E. Pflieger, Tatiana Stroganova

**Affiliations:** ^1^Department of Higher Nervous Activity and Psychophysiology, St. Petersburg State UniversitySt. Petersburg, Russia; ^2^Laboratory of Decision Making, National Research University Higher School of EconomicsMoscow, Russia; ^3^Complex Systems Control Laboratory, Institute for Problems of Mechanical EngineeringRAS, St. Petersburg, Russia; ^4^Laboratory of Cognitive Psychophysiology, National Research University Higher School of EconomicsMoscow, Russia; ^5^Dynamic Neuroimaging Laboratory, McConnell Brain Imaging Centre, Montreal Neurological Institute, McGill UniversityMontreal, QC, Canada; ^6^Source Signal Imaging Inc.San Diego, CA, USA; ^7^MEG Centre, Moscow State University of Psychology and EducationMoscow, Russia

**Keywords:** spatial components, ICA, SVD, components selection, mutual information, eloquent cortex mapping

## Abstract

Spatial component analysis is often used to explore multidimensional time series data whose sources cannot be measured directly. Several methods may be used to decompose the data into a set of spatial components with temporal loadings. Component selection is of crucial importance, and should be supported by objective criteria. In some applications, the use of a well defined component selection criterion may provide for automation of the analysis. In this paper we describe a novel approach for ranking of spatial components calculated from the EEG or MEG data recorded within evoked response paradigm. Our method is called Mutual Information (MI) Spectrum and is based on gauging the amount of MI of spatial component temporal loadings with a synthetically created reference signal. We also describe the appropriate randomization based statistical assessment scheme that can be used for selection of components with statistically significant amount of MI. Using simulated data with realistic trial to trial variations and SNR corresponding to the real recordings we demonstrate the superior performance characteristics of the described MI based measure as compared to a more conventionally used power driven gauge. We also demonstrate the application of the MI Spectrum for the selection of task-related independent components from real MEG data. We show that the MI spectrum allows to identify task-related components reliably in a consistent fashion, yielding stable results even from a small number of trials. We conclude that the proposed method fits naturally the information driven nature of ICA and can be used for routine and automatic ranking of independent components calculated from the functional neuroimaging data collected within event-related paradigms.

## 1. Introduction

Spatial decomposition is one of the key techniques applied to exploratory analysis of multichannel data in general, and to spatial-temporal electro- and magnetoencephalographic (EMEG) signals in particular. The most commonly used methods to obtain both spatial components and the corresponding temporal loadings are independent component analysis (ICA) (Comon, 1994), principal component analysis (PCA) (Golub and Van Loan, 1996) and factor analysis (FA) (Child, 2006).

The most frequently used approach for analysis of stimulus-locked averaged EMEG data is PCA, which can be performed using the singular value decomposition (SVD) of the stimulus-locked averaged data matrix (Lagerlund et al., 1997). This analysis is followed by thresholding the singular values (SV) spectrum to identify the subspace capturing the largest amount of data variance for a given approximation rank (Vandewalle, 1988). This technique is inherently power driven. Its application to the identification of the repetitive task-related signal subspace from the averaged ERP/F data relies on the assumption that the individual evoked responses are sufficiently well phase-locked to the stimulus. In that case, the stimulus-locked summation results in an enhanced relative power of the phase-locked component (Misulis, 1994).

**Figure 1:**
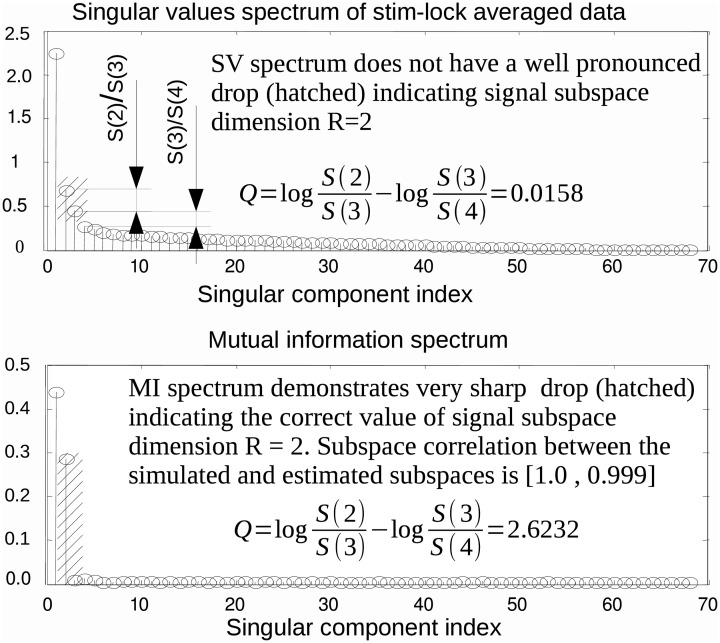
**In some practical cases, the first singular topographies remain reasonably good estimators of signal subspace but inspection of the SV spectrum fails to reveal this as illustrated by a model example here.** While the subspace spanned by the first two singular topographies and the actual simulated subspace practically coincide the SV spectrum (**top** panel) fails to reveal the fact that the second singular component also belongs to the signal subspace. On the contrary the MI spectrum (**bottom** panel) computed using raw data projected onto the left singular vectors demonstrates a very clearly cut separation of the task-related and task unrelated subspaces. This figure also introduces the measure of task-related subspace identifiability used in the paper. Since the correct signal subspace rank value is *R* = 2, we use discriminating indicator $q=log\left(\frac{S(2)}{S(3)}\right)-log\left(\frac{S(3)}{S(4)}\right)$ that formalizes the strategy employed by the human observers and estimates the amount of drop between the second and the third components referenced to the ratio of the two largest components of the noise range spectrum (with indices 3 and 4) immediately following the two signal components (with indices 1 and 2).

SVD is the optimal method for signal subspace detection measured by subspace correlation for a given approximation rank (Vandewalle, 1988). However, the actual value of signal subspace rank, *R*, is, in general, unknown. Finding an estimate of *R* is not a trivial task. It is often done by visual inspection of the SV spectrum. The method is based on identifying the target index, *R*, of a singular component just preceding a sharp drop in power, followed by a slow decaying plateau in the SV spectrum. However, a large disparity of activation amplitudes, spatial proximity of the neuronal sources and powerful noise sources may result in the absence of a clear cut division between task-related and noise components. In addition, in realistic conditions, the recordings are often contaminated by spatially colored brain activity and/or spatially coherent artifacts. Under these circumstances, component selection based on the SV spectrum may be misleading. As a motivating example, consider the top panel of Figure 1 that shows the SV spectrum calculated for the averaged data obtained from the simulated MEG timeseries containing the contribution of two non-synchronous dipolar sources. Although the subspace spanned by the first $\hat{R}$ = 2 singular topographies almost exactly matches the true subspace (subcorr([**a**_1_ **a**_2_], [**u**_1_ **u**_2_]) = [1, 0.987]) the spectrum of SVs fails to provide evidence that the second component contains task-related signal.

ICA is one of the most widely used approaches to blind source separation, popular for exploratory analysis of multidimensional data. In the analysis of EMEG data from evoked response experiments, this decomposition may be used both to isolate task-related components (Makeig et al., 1996; Vigario et al., 2000) and to remove artifacts (Jung et al., 2000). It can also be used for estimation of source timeseries when proper forward modeling is unavailable and for the estimation of the signal subspace in cases when the experimental paradigm can not guarantee sufficiently accurate stimulus locking, e.g., in voluntary movement paradigm (Ossadtchi et al., 2000; Delorme, 2010).

The application of ICA to routine analysis of EMEG datasets is limited by the absence of standard approaches for ordering the independent components (Hyvärinen et al., 2001). In the most typical scenario a human observer visually identifies the desired components by exploring their timecourses and topographies. Since the raw EMEG data are often very noisy, it can be difficult to determine which components should be selected. Additionally, such manual selection is often daunting, and selection based on power (Delorme, 2010) or stim-locked averaged power (AP) (Hyvärinen et al., 2001) does not ensure that the components are event-related for reasons essentially similar to those just described for the SV spectrum. It should be also noted that such power-driven ordering methods may be inappropriate, since ICA as an information (rather than power) driven technique. An alternative method, using the correlation metrics between each estimated component and the event trigger, is critically dependent on signal shapes, and is therefore highly unreliable. For completeness we will mention that for some methods of blind source separation, such as AMUSE (Tong et al., 1991), components may have an intrinsic order but such an ordering is not very useful in the context of analysis of EMEG data from ERP studies. These problems hinder efficient utilization of ICA for batch-mode processing of EMEG datasets, and affect the objectivity of the results obtained with manual analysis.

The independent components sorting problem has received considerable attention in the fMRI data analysis literature. Gu et al. (2001) and Esposito et al. (2002) have introduced methods for component ordering based on spatial characteristics. Lu and Rajapakase (2003) suggested ranking based on component timecourse kurtosis. Himberg et al. (2004) used clustering of a succession of ICA realizations to select relevant components. Yang et al. (2008) describes a method for components selection based on the reproducibility principle. In the application of ICA to EEG and MEG, a technique based on measuring the amount of spatial component variance explained by the electromagnetic model was proposed by Grosse-Wentrup and Buss (2008). However, an accurate forward model is required to fully benefit from this approach.

In the current paper, we present a novel mutual information (MI) based approach for ICA components sorting. Moritz et al. (2003) has described a somewhat related method for component ranking, based on the spectral manifestation of stimulus periodicity. However, the periodicity assumption is not always fulfilled, especially in voluntary movement paradigms. In addition, the spectral measure uses only first and second order statistical moments, while our MI-based method implicitly employs higher order moments for estimating the amount of task-related signal present in a component. We report the performance of our new MI based approach and compare it against more conventionally used AP driven technique. Additionally, we demonstrate an application of the MI Spectrum to sorting InfoMax ICA components obtained from real MEG recordings obtained from an experiment designed to non-invasively map primary motor cortex (M1 zone).

## 2. Materials and methods

### 2.1. EMEG signal model and preliminaries

EMEG data recorded by a *K* − sensor array during the *i*-th repetition of a neuromotor or cognitive task can be written as the following linear combination

(1) $$x^{i}(t)=\left[a_{1},\ldots ,a_{R}\right]\text{​}\left[\begin{array}{c} f_{1}^{i}(t)\\ \vdots \\ f_{R}^{i}(t) \end{array}\right]\text{​}+\left[b_{1},\ldots ,b_{L}\right]\text{​}\left[\begin{array}{c} p_{1}^{i}(t)\\ \vdots \\ p_{L}^{i}(t) \end{array}\right]\text{​}+n(t)\;$$

For the *i* − *th* epoch, a multichannel signal at each instance of time, **x**^*i*^(*t*) is a noisy additive mixture of source topographies [**a**_1_, …, **a**_*R*_] weighted by the corresponding stimulus-locked activation timeseries [*f*^*i*^_1_(*t*), …, *f*^*i*^_*R*_(*t*)], along with a task-unrelated contribution from sources with topographies [**b**_1_, …, **b**_*L*_] activated with task-unrelated timeseries [*p*^*i*^_1_(*t*), …, *p*^*i*^_*L*_(*t*)], and a random noise vector **n**(*t*). Topographies of task related sources form an *R*-dimensional signal subspace and topographies of task unrelated sources form an *L*-dimensional coherent interference subspace. ERP experiments are usually accompanied by a binary stimulus signal *s*(*t*) marking the task onset. In neuro-motor experiments this binary signal may be derived from the myographic activity record or from the accelerometer signal, using a thresholding procedure. Usually the goal of data analysis is to identify the task related signals and extract the task-related signal subspace to be used subsequently for neuronal source localization. For completeness, we may include induced sources whose activation power is locked to the task-onset moment with random phase. However, since we are interested in analysis of ERP's (which are phase-locked by definition), we do not include the induced component in (1).

Under the ideal and largely unrealistic conditions when activations *f*^*i*^_*r*_(*t*) are exactly reproducible across trials, time locked to the stimulus, and spatially coherent task-unrelated components are absent, the identification of task related components can be done optimally using the SVD of the stimulus locked average data matrix **X** = [**x**(0), …, **x**(*T*)], where $\bar{x}(t)=\sum_{i=1}^{M}x_{i}(t)$, *M* is the task repetitions count, and *T* is the interval of interest duration (Vandewalle, 1988). The SVD yields the averaged data matrix decomposition **X** = USV^*T*^. Columns of the orthonormal matrix **U** are the singular topographies, **S** is a diagonal matrix of SVs, and columns of the right singular matrix **V** are the singular activations. Task related components are chosen to be the first $\hat{R}$ components ranked by power. $\hat{R}$ is determined typically by visual analysis of the SV spectrum. Optionally, the SV spectrum of random matrix may be used as a reference in this task (Golub and Van Loan, 1996).

ICA is usually applied to the raw (unaveraged) spatial-temporal matrix **X**(*t*) and yields a spatial unmixing matrix **B** and a collection of independent components *z*_*i*_(*t*) obtained as **z**(*t*) = **BX**(*t*), **z**(*t*) = [*z*_1_(*t*), …, *z*_*K*_(*t*)]^*T*^. We assume that some of these components contain task-related signal and the others do not. In correspondence to **B** we can put matrix **F** = **B**^−1^ so that **X**(*t*) = **Fz**(*t*). Columns of **F** are called independent topographies and describe the profiles formed by the corresponding independent “sources” on the sensors.

### 2.2. Mutual information spectrum

We propose to assess the degree to which the *i*-th component *z*_*i*_(*t*) is related to the task using the normalized MI spectrum, computed as *I*_*i*_ = *I*(*z*_*i*_(*t*), *e*(*t*)). *e*(*t*) is the expanded stimulus line signal, computed by convolution of the original binary stimulus signal *s*(*t*) with expansion kernel *k*(*t*) as *e*(*t*) = *s*(*t*) ∗ *k*(*t*) to produce monotonic variations over the interval of interest around each event onset moment. In this work we used a centered (i.e., symmetric around the x-axis) ramp function as the expansion kernel *k*(*t*).

We used a simple scaled histogram method to compute the MI as the difference between the entropy of an independent component *z*_*i*_(*t*) and its entropy conditioned on the expanded stimulus line signal *e*(*t*), i.e.,

(2) $$I_{0}\left(z_{i}(t),e(t)\right)=H\left(z_{i}(t)\right)-H\left(z_{i}(t)|e(t)\right)$$

where *H*(*u*) denotes the entropy of *u*.

As suggested by Strehl, (2002), we use the geometric mean of the two marginal entropy values to obtain the normalized MI quantities as

(3) $$I_{i}=I\left(z_{i}(t),e(t)\right)=\frac{I_{0}\left(z_{i}(t),e(t)\right)}{\sqrt{H\left(z_{i}(t)\right)H\left(e(t)\right)}}$$

We then define the MI spectrum as the rank ordered elements *I*_*i*_ : {*I*_*i*_ ≥ *I*_*i* + 1_}.

As with the more conventional SV spectrum, visual analysis of the MI spectrum can be used to estimate the signal subspace rank. Originally suggested by us in Ossadtchi et al., (2000), this measure of MI with the expanded stimulus signal is a power-invariant way to assess the degree of task-relatedness of raw (unaveraged) timeseries.

We have introduced the notion of MI spectrum in the context of ICA component selection. This method can also be used for ranking singular components obtained via SVD of the stimulus locked average data matrix. To compute the MI spectrum for such singular components, first project the raw unaveraged data matrix **X**(*t*) onto the left singular column vectors of **U** as **z**(*t*) = **U^T^X**(*t*) and then apply the MI spectrum calculation procedure as described above.

In neuro-motor tasks, the EMG signal, *m*(*t*), can be recorded and used instead of the expanded stimulus line. Then the MI spectrum is calculated as *I*_*i*_ = *I*(*z*_*i*_(*t*), *m*(*t*)). Since the EMG signal usually occupies a broader spectrum than that of EEG or MEG signals, it is beneficial to perform a zero-phase-shift band-pass filter to remove excessively low and high frequency components prior to computing the MI.

### 2.3. Statistical testing

In automated applications, and for a more informed decisions during the visual analysis of the MI spectrum, we suggest the following randomization testing scheme to estimate the *p*-values to reject the null-hypothesis that a component is not task related.

The suggested scheme is based on the observation that for signal components that contain a statistically significant evoked response, the value of the MI is directly related to the consistent correspondence (not similarity!) between the shape of this component and the expanded stimulus signal. Therefore, when the actual task onset moments are randomized, this correspondence will be destroyed. The MI values for the task-related components will experience a significant drop, while those that pertain to the task-unrelated components will remain in the original range.

We suggest the following simple steps to generate surrogate data and assess statistical significance of the observed MI values. In what follows *M* is number of independent components, *J*-number of randomization iterations, *N*_*t*_ is the number of samples in the stimulus signal *s*(*t*) and $N_{r}=\sum_{t=1}^{N_{t}}s(t)$ is the number of task repetitions.

1. *for j* = 1:J
  1. Create a new, surrogate stimulus signal s^*^(*t*) by randomizing task onset moments:
    s^*^(*t*) = 0, ∀*t* ∈ [1, *N*_*t*_];
    for *k* = 1 : *N*_*r*_, *t* ← *U* (0, *N*_*t*_), s^*^(*t*) = 1, end_*k*_
  2. Calculate surrogate expanded stimulus signal: e^*^(*t*) = s^*^(*t*) ∗ *k*(*t*)
  3. Calculate the amount of normalized MI of all the components with this surrogate expanded stimulus:
    for *i* = 1 : *M*, I^*^_ij_ = *I* (e^*^(*t*), *z*_*i*_(*t*)), end_*i*_
2. end_*j*_

The *i*-th row of I^*^_ij_ measures the MI for the *i*-th component and the *j*-th randomization of the stimulus onset signal. In order to calculate the *p*-values of the null-hypothesis that the *i*-th component does not contain task-related signal, we compute the fraction of surrogate values I^*^_ij_, *j* ∈ [1, *J*] that exceed the actual observed value *I*_*i*_. If we define a logical function *L*(*a*, *b*) = 1 *if*(*a* > *b*) and *L*(*a*, *b*) = 0 otherwise, then *p*-values for the null-hypothesis that the *i*-th component contains no task related signal can be expressed as $p_{i}=\frac{1}{M}\sum_{j=1}^{j<J}L(I_{ij}^{*},I_{i})$.

### 2.4. Multiple comparison correction

**Figure 2:**
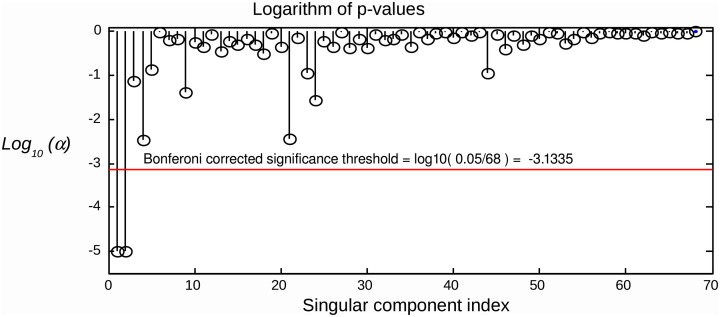
**Raw components' *log*(*p*)-values computed using the suggested randomization scheme.** The horizontal line corresponds to Bonferroni corrected threshold determined for α = 0.05. The data were simulated with two dipolar sources and powerful spatially coherent interference. SVD was applied to the stimulus-locked data matrix that yielded first singular topographies. Subspace correlation of the first two singular topographies with the true signal subspace was [1, 0.987]. We can see that the suggested randomization scheme is able to correctly detect the first two components that span the signal subspace.

Since we are testing several hypotheses, we need to correct the calculated *p*-values for multiple comparisons. Simple Bonferroni correction is appropriate, since the components are independent or at least orthogonal (SVD case). We therefore conclude that a component can be considered as task-related at the significance level of α if $p_{i}<\frac{\alpha }{N_{c}}$, where *N*_*c*_ is the total number of components tested. An example of applying the suggested statistical testing scheme is illustrated in Figure 2, where the proposed procedure allows for the correct identification of task related components in a simulated scenario with *R* = 2 task-related sources with powerful, spatially coherent, interference. For this and subsequent simulations, we used the procedure described in the following section.

### 2.5. Simulation procedure

**Figure 3:**
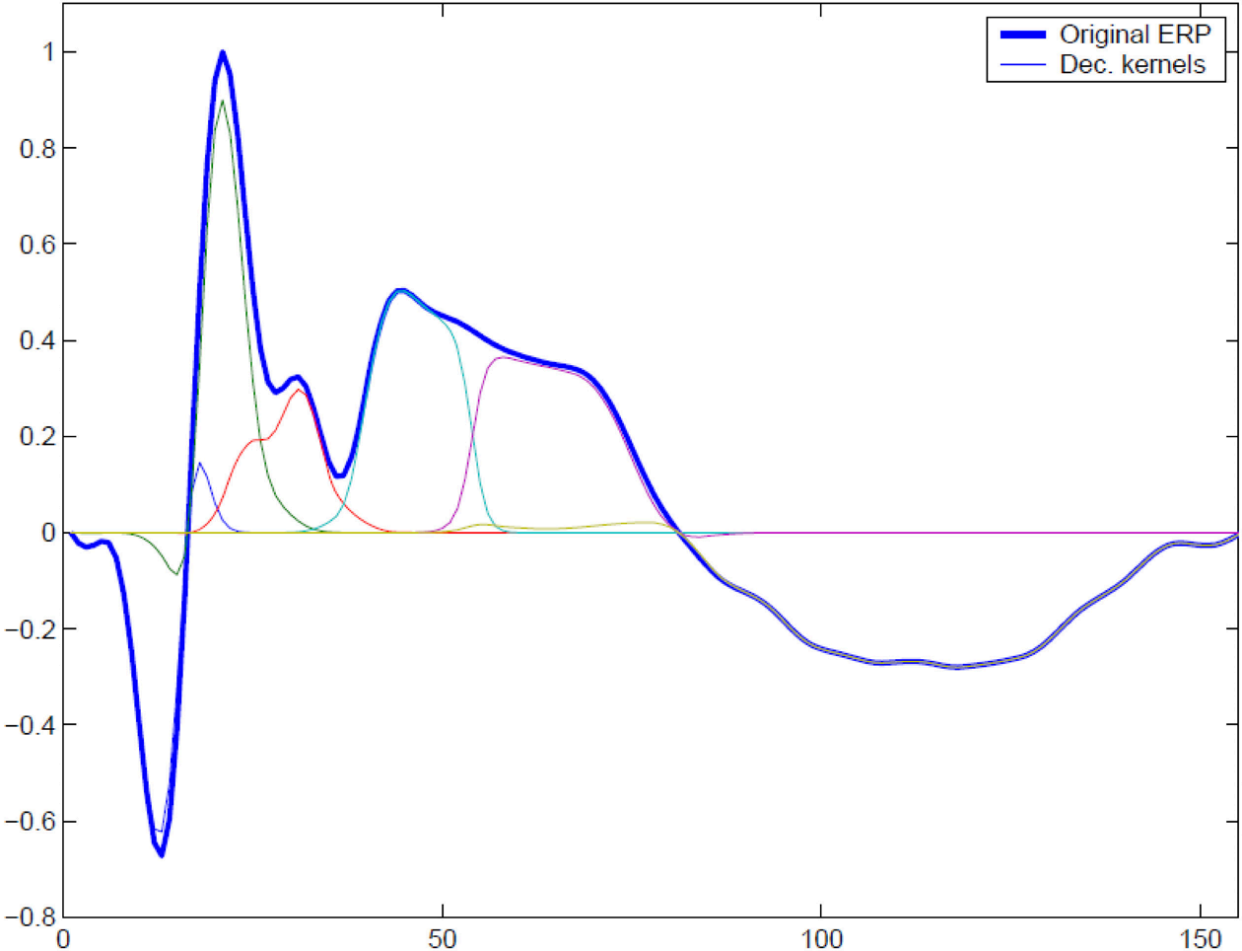
**To simulate trial-to-trial variation of the responses we used overlapping Gaussian kernel based model.** At each trial we varied relative positions of the kernel centers, kernel amplitudes, and global response latency with respect to the binary stimulus signal. A typical response and its representation with a set of overlapping modulated kernels is shown.

In order to illustrate the performance of the MI spectrum and compare it with a more standard power driven approach, we performed realistic simulations with the following procedure. To simulate the observed sensor signals, we used Equation (1). We obtained a realistic source configuration from analysis of a somatosensory MEG dataset recorded with a 67 channel CTF MEG system. We applied the RAP-MUSIC localization algorithm to [0–200 ms] range of the stimulus-locked average data and obtained *R* = 2 dipoles with topographies **a**_1_ and **a**_2_ and their corresponding activations *f*_1_(*t*) and *f*_2_(*t*) [see Equation (1)]. We used these dipoles as sources of task related activity in our simulations. To simulate task related activation timeseries we adapted a kernel-based model of evoked potentials described by Lange et al., (1997). This model includes random trial-to-trial variation in the latency and amplitudes of signal components. It is based on the decomposition of activation timeseries into a superposition of Gaussian kernels with varying amplitudes and delays. The model is justified by the fact that, given relatively poor spatial resolution of MEG, the dipole timeseries may be viewed as the sum of activations of several neuronal assemblies, each with different intensity and activation latency values. A graphical example of such a decomposition is shown in Figure 3. The simulated activation of the *r*-th dipole during the *i* − *th* epoch can be expressed formally as $f_{r}^{i}(t)=\sum_{k=1}^{K}\beta_{k}^{r}v_{k}^{r}(t-\theta_{k}^{r})$ with *K* kernels defined as

(4) $$v_{k}^{r}\left(t\right)=f_{r}(t)\frac{e^{-\frac{{\left(t-\tau_{k}^{r}\right)}^{2}}{2\sigma_{k}^{r2}}}}{\sum_{l=1}^{K}e^{-\frac{{\left(t-\tau_{l}^{r}\right)}^{2}}{2\sigma_{l}^{r2}}}},\;r\in \left[1,R\right],\;k\in \left[1,K\right]$$

The model incorporates random variables β_*k*_, θ_*k*_, *k* = {1, …, *K*} representing amplitude and latency variations. The latency jitter values were independent for all components and were generated using a Gaussian random variable with mean of 50 ms and standard deviation 10 ms.The *k*-th kernel amplitude variation β_*k*_ was modeled as normally distributed random variable with mean of unity and standard deviation equal to 0.2.

We modeled brain noise with *L* = 1000 spatially coherent, task-unrelated cerebral sources whose locations and time series varied with each realization. The corresponding topographies **b**_*l*_ were calculated using locally fitted concentric spheres MEG forward model as implemented in EMSE Software Suite, Source Signal Imaging Inc., San Diego, CA, USA. The activation time series were narrow-band signals obtained via zero-phase filtering of realizations of Gaussian (pseudo)random process by the fifth order band-pass IIR filters in the bands corresponding to theta (4–7 Hz), alpha (8–12 Hz), beta (15–30 Hz) and gamma (30–50 and 50–70 Hz) activity. Their relative contributions were scaled in accordance with $\frac{1}{f}$ characteristic of the realistic EMEG spectrum. An additional narrow-band alpha-component (9–11 Hz) of occipital origin ([−0.05, 0.01, 0.06] in EMSE coordinate system) was also included. We scaled the brain noise components to match typical signal-to-noise ratio of real-life recordings.

## 3. Results

### 3.1. Discriminative power of the MI spectrum

Consider the situation when a task-related signal is generated by a pair of dipolar sources. When a pair of sources has highly correlated topographies or, in case of a large imbalance in source magnitude, the second singular component may be obscured and may not produce a pronounced SV distinguishable from the baseline. In this case, analysis of the SV spectrum will fail to provide the correct estimate of the signal subspace dimension. An example is illustrated in Figure 1, where the SV spectrum of the averaged data matrix obtained from a simulated dataset with *R* = 2 task-related sources does not have a significant drop between *R* = 2 and 3. On the contrary, the MI spectrum exhibits a very clear separation between task-related and task-unrelated parts, as it can be seen in the bottom panel of Figure 1.

**Figure 4:**
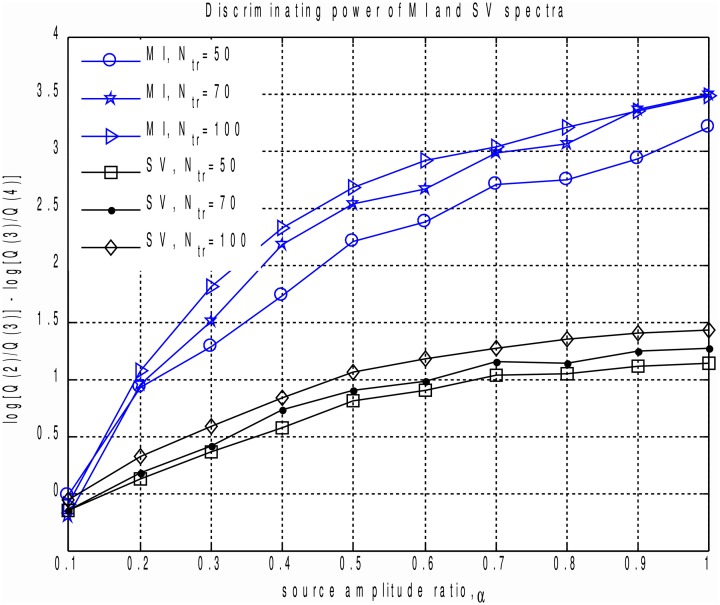
**Discriminating power indicator *q* as a function of source amplitudes ratio for different number of trials calculated for MI and SV spectra.** Each curve corresponds to a fixed datapoints count. We can see that the proposed MI based measure outperforms power based technique and produces a clearer cut between the task-related and task-unrelated components.

In order to perform a more systematic evaluation of using MI to measure the extent to which a component is task-related, we performed a set of simulations with two dipolar sources in the presence of realistic brain noise. We varied the ratio of activation amplitudes of the two dipoles, performed SVD of the averaged data matrix and calculated the MI spectrum for the projections of continuous data onto the left singular vectors. We then compared the discriminating power of the MI and the SV spectra. To do so we introduced the discriminating indicator *q*. Since the correct rank value is *R* = 2 we used $q=log\left(\frac{S(2)}{S(3)}\right)-log\left(\frac{S(3)}{S(4)}\right)$, see Figure 1. *q* is sensitive to the drop between the second and the third components, referenced to the ratio of the two largest noise range spectrum values (with indices 3 and 4) immediately following the two signal components (with indices 1 and 2). Results are shown in Figure 4, illustrating the discriminating indicator *q* as a function of source amplitudes ratio. We performed this numerical experiment for a varying number of trials in a simulated dataset. We found that the proposed MI spectrum outperforms the SV spectrum for all trial counts, and also provides for a clearer seperation between the task-related and task-unrelated components. Also note that in most cases the correlation of the subspace spanned by the first two singular topographies and the true signal subspace spanned by **a**_1_ and **a**_2_ was sufficiently high to be considered as a correct estimate of the simulated signal subspace.

### 3.2. Receiver operating characteristics of MI

In this section we describe our experiments on exploring receiver operating characteristics (ROC) of the MI metrics. We consider the task of discriminating between the components that contain task-related signal and those that do not. Spatial components *z*_*i*_(*t*) obtained from signals that can be represented using Equation (1) can be viewed as the superposition of signal and noise, expressed as

(5) $$z(t)=af(t)+\sigma_{p}p(t)+\sigma_{n}n(t),$$

where *f*(*t*) is task-related source activity of amplitude *a*. *p*(*t*) and *n*(*t*) are the contributions from spatially coherent and spatially white noise sources, with standard deviation values σ_*p*_ and σ_*n*_ respectively.

We simulated repetitions of the task-related signal, including the jitter and variations characteristic of realistic brain signals. For each of *N*_*mc*_ = 1000 Monte-Carlo realizations, we simulated *N* = 100 signals according to Equation (5). *N*_1_ = 10 out of 100 signals had *a* = 1 and the remaining *N*_0_ = 90 signals had *a* = 0, i.e., no task-related signal present. We simulated brain noise as described in the Simulations Procedure section. The goal was to detect the components that contain task related signals. We compared the MI values against the more traditionally used stimulus-locked averaged signal power (AP), calculated as

(6) $$P_{i}=\sum_{t\in W}{\bar{z}}_{i}^{2}(t),$$

where *z*_*i*_(*t*) is the stimulus-lock averaged *i*-th signal. The summation was performed over a 200 ms window centered ont the stimulus. We used the same windows to calculate both MI and AP measures.

To calculate the ROC curves, we applied thresholding to the MI and AP spectra separately, and marked as detected only those components whose corresponding MI or AP values exceeded the threshold. The threshold was originally chosen to be 0.05 of the largest value in the spectrum (AP or MI). In order to obtain the ROC curve we calculated the sensitivity $p_{sens}(\theta )=\frac{\text{NTP}}{N_{1}}$ and specificity $p_{spec}(\theta )=1-\frac{\text{NFP}}{N_{0}}$ for a succession of evenly spaced threshold values θ = 0.05 *k* max_*i*_(*I*_*i*_) or θ = 0.05 *k* max_*i*_(*P*_*i*_) for *k* = 1, …, 19.

**Figure 5:**
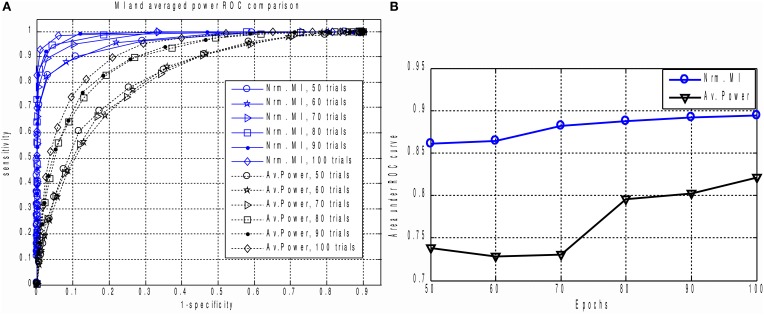
**(A)** The family of ROC curves for Averaged Response Power and Mutual Information based detection for various number of epochs on the same plot. MI based detection clearly and significantly outperforms the conventional method. Even with 50 trials the MI based criterion (“Nrm. MI, 50 trials” curve) allows to achieve 70 percent of sensitivity with ideal specificity. We can see that for all counts of epochs the MI based measure significantly outperforms the power based characteristic. This can be explained by the fact that in calculation of MI we implicitly take into account higher order information as compared to the power based approach where only the first two statistical moments are used. **(B)** Area under ROC curve performance characteristic for Averaged Response Power and Mutual Information.

The result is shown in Figures 5A,B. For all epoch counts, the MI based measure significantly outperforms the power-based characteristic, and also provides better sensitivity for any selected specificity. We have observed similar behavior when dealing with real MEG data, as described below in the “MI versus AP for small epoch counts in real data” section. The improved ROC may be explained by the fact that the MI based measure implicitly takes into account higher order statistical information as compared to the power based approach, where only the first and second order statistical moments are used.

### 3.3. Application to M1 mapping

Reliable mapping of the primary motor cortex (M1) based on functional neuroimaging provides an important complement to the use of structural data alone. However, since various zones forming the somatosensor complex appear to be in a coupled interaction even in the motor planning stage, the localization of M1 zone from the functional EEG and MEG data via standard approaches is problematic and often does not yield reliable results (Sanders et al., 1996; Gerloff et al., 1998).

Inspired by the work of Riehle (2005) we explored the possibility of using the information from activation timecourse morphology and looked for spatial properties of activations with sharp non-linear increase just preceding the movement onset. To do so we studied MEG-recorded brain responses during a voluntary index finger movement task performed by 18 healthy right-handed volunteers.

**Figure 6:**
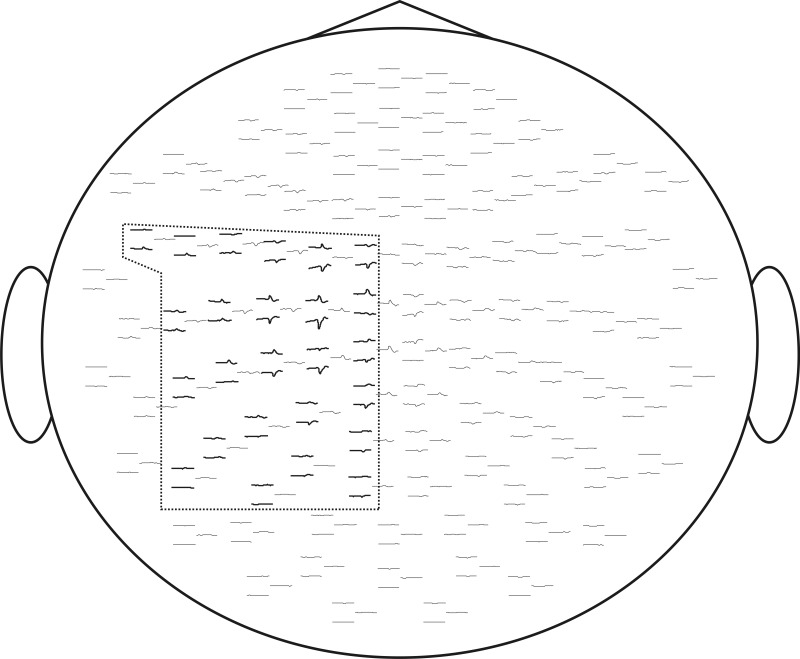
**We selected for ICA analysis 50 electrodes over the left sensory-motor cortex corresponding to the cortical representation of the right hand.** Evoked response fields are shown. The selected sensors are framed.

For computational feasibility we used a subset of 50 sensors located over the left sensory-motor region. These were selected based on the grand-average responses, as shown in Figure 6. Recordings from all experimental sessions in all subjects were concatenated into a single sequence and decomposed using the InfoMax-ICA approach. We obtained 50 independent components and ranked them according to the amount of MI with the expanded stimulus signal *e*(*t*) = *s*(*t*) ∗ *k*(*t*) in the 200 ms window centered around the movement onset moment marked by *s*(*t*). The choice of the time window is motivated by our interest in the early components reflecting the activity M1 zone.

**Figure 7:**
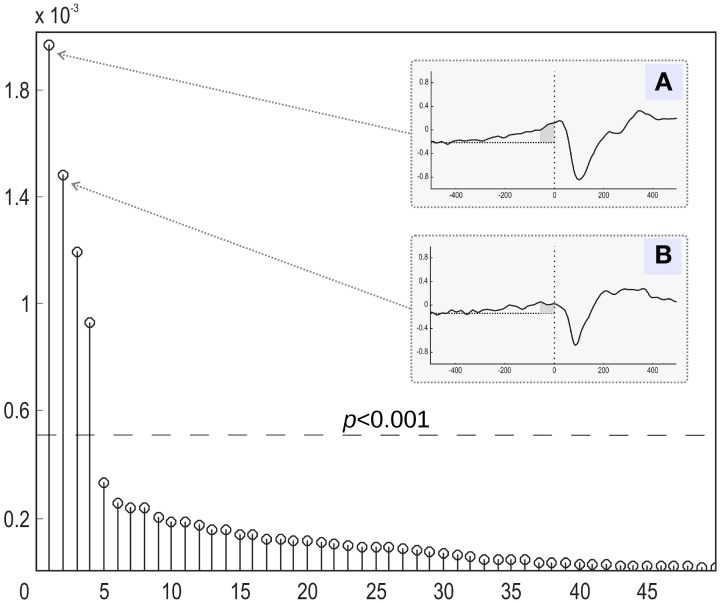
**Relative contribution of independent components of the evoked magnetic activity accompanying movement onset.** All derived components (abscissa) are ranked according to the amount of mutual information with the expanded stimulus signal. The MI values were normalized to the overall amount of information (ordinate). **(A,B)** The stimulus-locked average time courses of the two components (4 and 1) with the greatest share of mutual information with expanded stimulus signal. Abscissa: time (ms) in relation to the movement onset. Ordinate: component amplitude (arbitrary units). Low-pass filter with 30 Hz cutoff frequency was applied. Movement onset is shown by the vertical dotted line. Horizontal dotted line represents the background signal level. Dark-gray area shows time interval of exponential growth preceding movement onset of component 4.

We then focused on the first two components with the largest mutual information, as shown in Figure 7. The temporal dynamics of the first two components showed a slow activation increase starting as early as 400 ms before the actual movement onset. However, as illustrated in Figures 7A,B, the two components differed in their behavior during the interval directly preceding the movement onset. The component with the larger value of MI exhibited a sharp quasi-exponential growth starting at around 50–70 ms before the movement onset. The onset dynamics of the second component was smooth, corresponding to a quasi-linear growth. After movement onset both components show a pronounced negative deflection reaching a minimum at around 100 ms after the movement onset.

**Figure 8:**
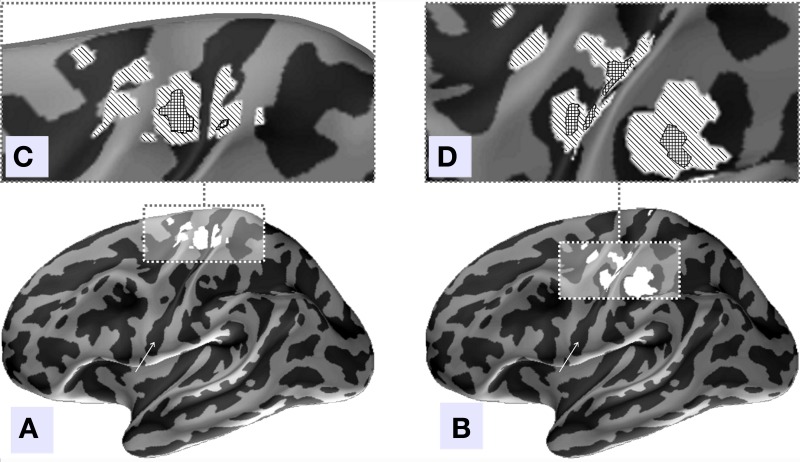
**Source localization of the two independent components with the largest amount of MI, (A) component 4, (B) component 2.** Inflated surface of the left hemisphere obtained by averaging of the co-registered individual cortices of 18 subjects is shown. Light-gray areas represent gyri and dark-gray areas represent sulci. The central sulcus is indicated by arrows. Localization of the sources of both components was done via MNE approach followed by Bonferroni correction. **(C,D)** Illustrated patches correspond to current distribution thresholded at significance level of *p* < 0.001. **(C)** We can see that in agreement with our hypothesis the topography of the independent component with the largest amount of MI predominantly localizes anterior to the central sulcus and may represent the M1 zone. **(D)** The second component's sources localize to the post-central fissure and in the depth of the central fissure inferior to the omega shaped zone.

We used MNE distributed source imaging to localize the neuronal generators underlying the topographies of the first two components with the highest values of MI. In both cases we observed activations in the area surrounding the central fissure of the hemisphere contralateral to movement, shown in Figure 8. After thresholding at *p* < 0.001 we observed that cortical areas subserving these two components do not overlap, as shown in Figure 8. Cortical sources of the first component localized primarily on the anterior slope of the central sulcus superior to the omega zone, shown in Figure 8C. This source most likely lies in M1, based on the anatomy. The cortical sources for the second component were located in the post-central sulcus and in the depth of the central sulcus inferior to the omega shaped zone, shown in Figure 8D.

### 3.4. MI versus AP for small epoch counts in real data

**Figure 9:**
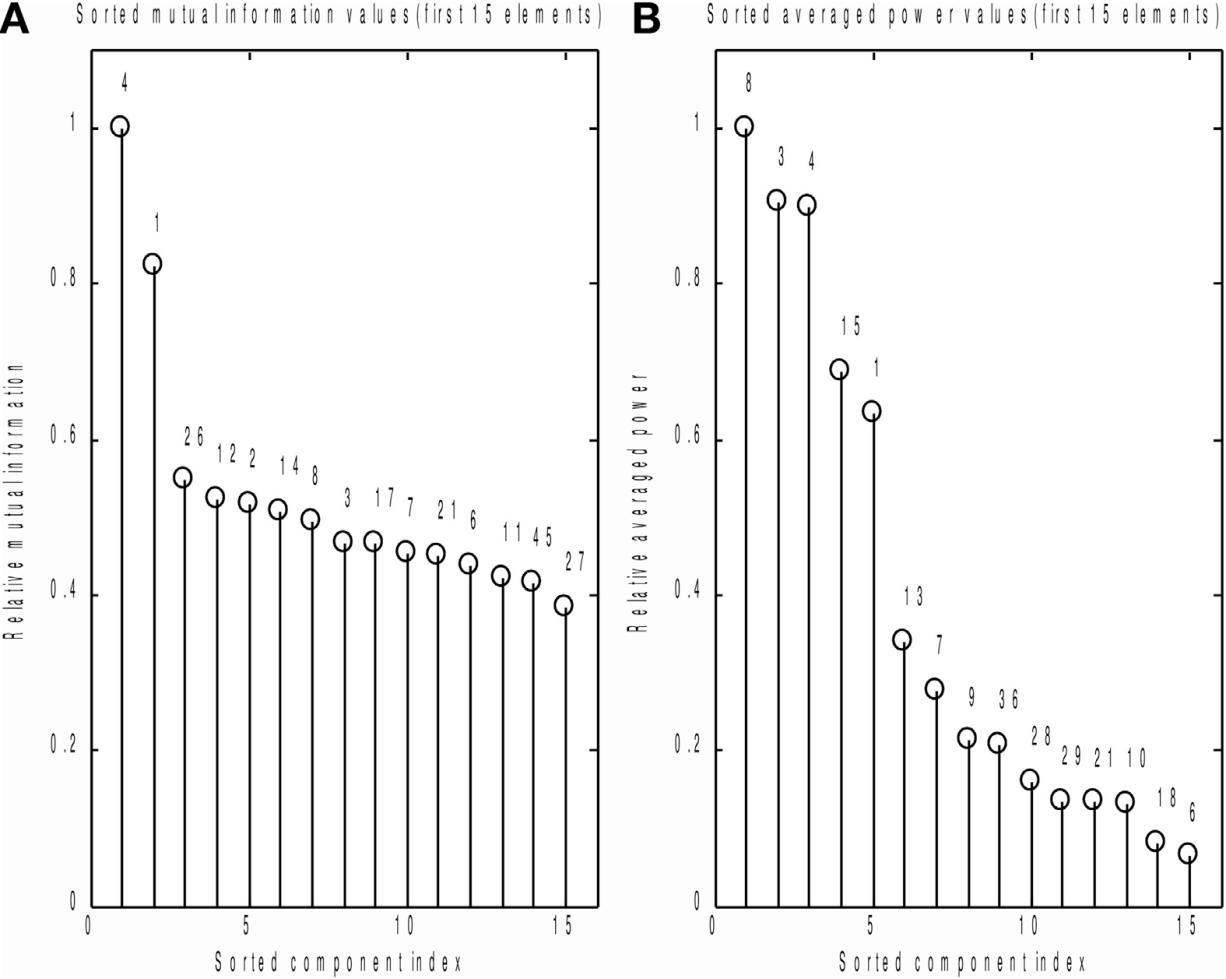
**Analysis of a subset of trials.** Normalized by the first element value MI **(A)** and AP **(B)** spectra calculated using a subset of events for the first 15 independent components. On the top of each stem actual (unsorted) component number. As prescribed by the MI spectrum we should select components 4 and 1 as task related. AP spectrum suggests components 8, 3, 4, 15, 1 for this role. In order to check which of the two measures provide the right answer we inspected stimulus-locked averages of the components suggested by the two measures, see Figure 10.

We also compared the performance of the MI spectrum with the more conventional AP metric (6) when the number of epochs is limited. We took every 30th event and analyzed the data according to the scheme described above. Independently sorted MI and AP spectra for the first 15 components are shown in Figures 9A,B respectively. The MI spectrum clearly shows the presence of task-related signal in the first two components with original indices 4 and 1. The AP spectrum shows five seemingly task-related components (indices 8, 3, 4, 15, 1) standing out from the baseline. Components 1 and 4 are identified as task-related by the both measures of task-relatedness.

**Figure 10:**
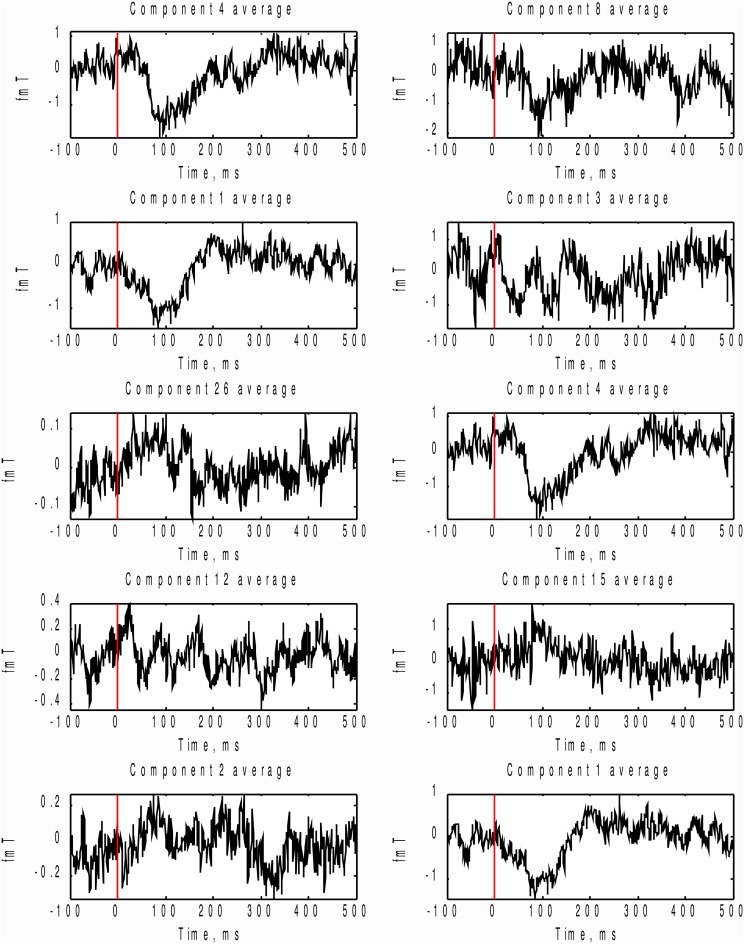
**In order to check which of the two methods provide the right answer we performed stimulus-locked averaging of the first five components in the order prescribed by sorting of MI and AP spectra (Figure 9). Left** panel corresponds to MI prescribed components and the **right** panel - to AP. As it can be seen the first two components (4 and 1) emphasized by the MI spectrum (Figure 9A) show a clear task related deflection, the subsequent components do not have significant amount of stimulus-locked activity and therefore are most likely unrelated to the task as correctly indicated by the characteristic drop in the MI spectrum (Figure 9A). Three out of five (8, 3, 15) component averages prescribed by the AP spectrum do not exhibit the expected deflection.

In order to check which of the two methods provided the correct answer, we performed stimulus-locked averaging of the first five components obtained by sorting in decreasing order the MI and AP spectra, shown in Figure 9. The results are shown in Figure 10. The first two components (4 and 1) identified by the MI spectrum (see Figure 9A) show a clear task related deflection. The remaining components do not have significant amount of stimulus-locked activity and therefore are most likely unrelated to the task. Three out of five components identified by the AP spectrum (first two and the fourth) do not exhibit any deflection resulting from coherent summation. Note also that components 1 and 4 are among the five components selected by the AP spectrum (the third and the fifth). Visual analysis of the averages obtained for the other three components suggested by the AP spectrum does not reveal the presence of significant amount of stimulus-locked activity in three out of five components.

Based on these observations, we conclude that both MI and AP spectra demonstrate identical sensitivity, as both were able to detect two clearly task-related components (1 and 4) that were also found using the full dataset and characterized by inverse modeling (see Figure 8). However, the MI spectrum exhibits optimal specificity, identifying two components (Figure 9A). Both of these components appear to have a task-related deflection in their stimulus-locked averaged profiles (Figure 10A). The specificity of AP based measure r is poor by comparison with the MI measure, since AP it identified 5 components (Figure 9B), including 3 false positives and 2 correct hits (Figure 10B). The observed behavior is consistent with our simulation studies, illustrated in Figures 5A,B, where the MI spectrum demonstrated significantly higher ROC characteristics, and provided higher specificity for any fixed sensitivity value compared to the AP spectrum.

## 4. Discussion

We describe a novel information-theoretic approach for spatial components ranking. Our method is based on the MI Spectrum which serves as a power-invariant measure of repetitive task-related signal in the temporal loadings of spatial components. Using realistic simulations we demonstrated that the task-relatedness measure, based on estimating the MI between a component and the expanded binary stimulus signal, allows for significantly higher detector characteristics when compared with conventional alternatives. It also provides a means for more clear-cut separation of task-related and task-unrelated components when compared with the standard power driven approach that is used in SVD, and sometimes used for ranking ICA components as well. The MI measure can be used for sorting the components obtained from any sort of spatial decomposition, as long as it is possible to calculate the quasi-continuous timeseries underlying the components of interest. The demonstrated advantage in performance over the power-driven measure makes the MI spectrum method a candidate for the routine use in ranking both SVD and ICA components in the analysis of ERP data. Since the MI method is insensitive to powerful non-task-related noise sources, it should also facilitate automatic unsupervised analysis of ERP data using ICA.

The method can be easily extended to extract not only the evoked (phase-locked to the stimulus) activity but also band-specific task induced activity that is characterized by random phase but stimulus-locked power fluctuations. Such an extension would require that the band-pass filtered components envelope should be calculated before MI spectrum estimation.

We have also investigated the MI method performance applied to an MEG dataset in a voluntary finger movement task. Such paradigms present special challenges, since they include large amount of random latency jitter when compared with an external stimulus driven paradigms. This increased jitter comes from the inevitable errors in the estimates of motion onset obtained from the accelerometer signal. Nevertheless, the MI measure supported a clear cut separation of four task related components (Figure 7). The component with the largest MI (index 4) demonstrated a non-linear increase of activation just prior to the motion onset. In agreement with the previous experiments on primates (Riehle, 2005), this component localized primarily to the anterior slope of the central sulcus superior to the omega zone (Figure 8C), and most likely originates in M1. Thus, we demonstrated the potential to localize M1 non-invasively on a group level, using a functional probe.

In order to compare the performance of the MI and power based measures on the experimental dataset, we used a reduced number of trials. As shown by simulations (Figures 4, 5), this reduction should increase the contrast between the performance characteristics of the two methods. It should also mimic more realistic scenarios, when only a single subject dataset is used for ICA analysis. Under these conditions, we demonstrated that the proposed MI based measure for a fixed sensitivity value yields significantly higher specificity than the more conventional power based measure. This result is in agreement with our simulation studies.

In the current work we used a simple histogram-based approach for calculation of MI, omitting any bias correction. For realizations of independent random processes Treves and Panzeri (1995) have shown that MI estimate bias is quadratically proportional to the number of histogram bins used to approximate the pdf of continuous random processes and is inversely proportional to the number of datapoints. Since we used a large number of datapoints (*N* ≈ 6.5 × 10^5^) in our experimental data analysis compared to the *K* = 10 bins used for approximation of the probability density functions, we do not expect a bias correction procedure to appreciably alter the observed MI. However, it has also been shown (Chrisman, 2013) that the bias decreases as the true MI between the timeseries pairs grow. This means that bias may result in MI values of task-unrelated components being overestimated, yielding a decreased contrast between the task-related and task-unrelated components in the MI spectrum. In our simulation studies we used a relatively small number of datapoints compared to a standard EEG/MEG data recording per single patient. Therefore, we expect that the observed performance (Figures 4, 5) may be further improved with a proper bias correction procedure. The use of a biased estimator in the statistical testing approach we implemented results in less sensitive tests, since the null-hypothesis distribution estimate appears to be “shifted to the right.” Selection of an appropriate bias correction method, however, requires a significant amount of additional numerical experiments and goes beyond the scope of this paper.

### Conflict of interest statement

The authors declare that the research was conducted in the absence of any commercial or financial relationships that could be construed as a potential conflict of interest.
